# The Influence of Vitamin D Levels on Dental Caries: A Retrospective Study of the United States Population

**DOI:** 10.3390/nu16111572

**Published:** 2024-05-22

**Authors:** Man Hung, Himani Patel, Samantha Lee, Justin Nguyen, Amir Mohajeri

**Affiliations:** 1 College of Dental Medicine, Roseman University of Health Sciences, 10894 S. River Front Parkway, South Jordan, UT 84095, USA; 2 Division of Public Health, University of Utah, Salt Lake City, UT 84108, USA; 3 Department of Orthopaedic Surgery Operations, University of Utah, Salt Lake City, UT 84108, USA; 4 George E. Wahlen Department, Veterans Affairs Medical Center, Salt Lake City, UT 84148, USA

**Keywords:** vitamin D, dental caries, oral health, NHANES, socioeconomic factors, vitamin D deficiency, dental health

## Abstract

(1) Background: This study examines vitamin D’s impact on dental caries to inform prevention strategies, given its critical role in bone and calcium regulation, vital for dental health. (2) Methods: Data from 18,683 participants of the National Health and Nutrition Examination Survey (NHANES) 2011–2016 were analyzed. NHANES collects U.S. population data through interviews, physical exams, and tests, including vitamin D levels and dental health assessed using both the decayed, missing, and filled teeth (DMFT) index and the presence of untreated dental caries. Vitamin D levels were measured according to serum 25(OH)D concentrations, and the analyses adjusted for confounders such as body mass index (BMI) and socioeconomic status (SES) using Chi-square, Mann–Whitney U, Kruskal–Wallis tests, as well as logistic and Poisson regression. (3) Results: This study found a mean DMFT score of 7.36 and a 33.2% prevalence of untreated dental caries. A higher caries prevalence was correlated with a lower SES (*p* < 0.001), the male gender (*p* < 0.001), and a higher BMI (*p* < 0.001). Severe vitamin D deficiency (<25 nmol/L) doubled the risk of dental caries, with odds ratios of 2.261 and 1.953 after adjusting for demographic factors and BMI. (4) Conclusions: Our study confirms a significant relationship between low vitamin D levels and an increased risk of dental caries nationwide, even after accounting for sociodemographic factors, emphasizing the importance of maintaining sufficient vitamin D levels for preventing caries.

## 1. Introduction

Vitamin D, a fat-soluble nutrient, is essential for maintaining overall health, particularly in bone metabolism and immune function. It is synthesized in the skin through sunlight exposure and is also obtained from dietary sources and supplements [1]. Vitamin D plays a key role in calcium homeostasis, crucial for optimal skeletal health. Since the early 1930s, the significance of vitamin D in preventing rickets and promoting bone health has been recognized [2]. Vitamin D facilitates the active absorption of calcium from the small intestine [3], which combines with phosphorus to form hydroxyapatite crystals, thus strengthening bones. Therefore, a diet rich in both vitamin D and calcium is vital for bone mineralization [3].

The link between vitamin D and dental caries has recently gained attention due to its implications for oral health. Dental caries, or tooth decay, is a prevalent chronic condition affecting individuals across all age groups [4]. This condition can manifest aggressively in early childhood, affecting primary teeth in infants and toddlers [5]. Factors influencing the risk of caries include the presence of cariogenic bacteria, low salivary flow, inadequate fluoride exposure, poor oral hygiene, inappropriate infant-feeding practices, and socioeconomic challenges [5].

In the United States, annual dental visits have been reported to be the lowest among individuals with lower education and income [6]. This plays a significant role in the formation of dental caries as there is a lack of preventative measures including oral hygiene instructions, fluoride varnish placement, and nutritional counseling [7]. Families with lower education and income are vulnerable to consuming a highly processed diet with a high sugar intake [8]. This type of diet results in deficiencies in essential vitamins such as vitamin D [9]. Refined sugars contain 99% sucrose, which is found primarily in highly processed foods [10]. Sucrose promotes the accumulation of Streptococcus mutans, a disruptive bacteria known for its ability to ferment sugars into acid [11]. This reduces the pH of the oral cavity, enhancing the demineralization of the enamel and leading to the progression of dental caries [12]. Additionally, poor oral hygiene, unhealthy eating habits (including eating disorders), and changes in the oral bacterial flora are risk factors for cavities [13]. The oral hygiene habits of parents have also been shown to influence those of their children, including the duration, frequency, and method of brushing as well as the most common sites brushed [14]. Policies and resources are needed to improve access to dental care through dental insurance that reduces out-of-pocket costs and through the integration of oral and medical health care [15].

Emerging research suggests a strong correlation between vitamin D levels and the incidence of dental caries [16,17]. One systematic review of controlled clinical trials indicated that vitamin D supplementation could reduce caries risk by 47% [18]. Deficiencies in vitamin D correlate with a reduced activity of antibacterial peptides such as cathelicidins and defensins, decreased saliva secretion, and lower calcium levels in saliva [19,20]. Thus, sufficient vitamin D levels may protect against caries by promoting tooth mineralization, enhancing immune responses, and modulating inflammatory processes [21]. Additionally, vitamin D is crucial for maintaining and utilizing calcium, which is necessary for normal salivary fluid and electrolyte balance in the parotid gland [22]. Factors such as a low salivary flow rate and high saliva viscosity are associated with an increased caries risk [23]. 

While numerous studies have focused on the preventive role of vitamin D supplementation in dental caries [18,24], broader factors contributing to caries development beyond vitamin D levels are often neglected. A comprehensive understanding of the interplay between vitamin D and biological, environmental, and behavioral determinants is crucial for developing more effective preventive interventions [25,26]. This study aims to address these gaps by further exploring the influence of vitamin D on dental caries, highlighting potential avenues for intervention and management.

## 2. Materials and Methods

### 2.1. Data Source 

This retrospective study utilized data from three cycles (2011–2016) of the National Health and Nutrition Examination Survey (NHANES). The NHANES employs a multilevel probability sampling method to collect data biennially from a representative sample of the U.S. non-institutionalized civilian population, spanning all age groups and residing across all 50 states and Washington D.C. We focused on post-2011 data because the NHANES includes a race/ethnicity variable for Asian people and detailed dental caries data for more accurate analysis. The survey includes interviews, physical exams, and laboratory tests, incorporating health metrics such as body mass index (BMI) and oral health evaluations, ensuring concurrent assessments within each cycle. This methodology aligns with the survey’s broader objective to examine health correlations, including those between oral health and other health indicators. Approximately 10,000 individuals participate per cycle, with response rates of 72.6% in 2011–2012, 71.0% in 2013–2014, and 61.3% in 2015–2016 [27]. Ethical clearance was granted by the National Center for Health Statistics Research Ethics Review Board (Protocol #2011–17 and its continuations). Consent was obtained in writing from the parents of minor participants. Detailed NHANES information is available on the Centers for Disease Control and Prevention website [28]. 

In this study, we included participants who had complete data on both their vitamin D levels and oral health examinations, totaling 23,151 individuals. After excluding 4468 participants due to them having incomplete data on other relevant factors, the final sample comprised 18,683 participants.

### 2.2. Measures 

Dental caries assessments were conducted by licensed dentists trained in NHANES-specific protocols using specialized equipment at the Mobile Examination Center (MEC). We used two primary indicators: the decayed, missing, and filled teeth (DMFT) index, and the presence of untreated dental caries. Untreated dental caries was identified by observing at least one tooth surface with a condition score from 0 to 4, or any untreated carious root tips [29]. 

Independent variable vitamin D levels were measured as continuous variables representing serum 25(OH)D concentrations, combining 25(OH)D2 and 25(OH)D3 levels, as determined using ultra-high-performance liquid chromatography–tandem mass spectrometry [30]. Vitamin D deficiency (VDD) was categorized as severe VDD (<25 nmol/L), moderate deficiency (25–50 nmol/L), insufficiency (50–75 nmol/L), or sufficiency (>75 nmol/L) [31,32,33,34]. 

### 2.3. Covariates 

We considered several potential confounders, including demographic characteristics, BMI, added sugar consumption, and socioeconomic status (SES), assessed using the poverty income ratio (PIR) [35,36]. PIR categories were defined as low-income (PIR ≤ 1.3), middle-income (PIR > 1.3–3.5), and high-income (PIR > 3.5) [37]. The participant demographic factors we included were age, sex, race or ethnicity (Mexican American, Other Hispanic, Non-Hispanic White, Non-Hispanic Black, Non-Hispanic Asian, or Other Race—including multi-racial individuals), and the family educational level (<9th Grade, 9th–11th Grade, High-School Graduate or General Education Development (GED), Some College, and College Graduate or Higher). Utilizing data from the NHANES, which included dietary intake information collected over a 2-year period via 24-hour recall interviews at the MEC, the added sugar consumption among participants was estimated using the United States Department of Agriculture’s (USDA’s) Food Patterns Equivalent Database (FPED) [38]. 

We utilized the FPED from the USDA to estimate the added sugar consumption for individuals in each NHANES cycle [39]. This database reports the content of added sugars in teaspoon equivalents (tsp. eq.), where one tsp. eq. is defined as 4.2 g of sugar, i.e., the amount found in one teaspoon of granulated sugar. According to the FPED, added sugars include sugars, syrups, or caloric sweeteners added during food and beverage processing or preparation at home, as well as sugars added at the table, such as in coffee or tea [40]. Participant body measurements were conducted at the MEC by trained health technicians. Height was measured using a stadiometer with an adjustable headpiece and a fixed vertical backboard, and weight was measured using a digital scale with participants wearing the standard MEC examination gown [41]. BMI levels were categorized into three groups: underweight (BMI < 18.5), normal weight (BMI 18.5–25), and overweight (BMI > 25) [34].

### 2.4. Statistical Analyses 

Between-group distinctions were evaluated employing the Chi-square test for categorical variables, while the Mann–Whitney U test was used to compare two groups, and the Kruskal–Wallis test was utilized to compare more than two groups for continuous variables. Dunn’s multiple comparison post-hoc test was followed when the omnibus results of the Kruskal–Wallis test were significant.

Logistic regression models assessed the association between vitamin D levels and untreated dental caries, expressed as odds ratios with 95% confidence intervals. Poisson regression was used to evaluate the relationship between vitamin D levels and the caries experience, with the results being presented as rate ratios (RRs). The regression analysis commenced with Model 1, which assessed the basic relationship between vitamin D levels and untreated dental caries. Model 2 expanded this by adjusting for sociodemographic variables such as sex, age, race/ethnicity, the PIR, and the family educational background. In Model 3, additional adjustments were made for the participants’ consumption of added sugars and their BMI. These three sequential models were utilized to evaluate the association between vitamin D levels and the presence of dental caries. Statistical significance was determined when the two-sided *p*-value was less than 0.05.

## 3. Results

Data from 18,683 participants spanning the NHANES cycles of 2011–2012, 2013–2014, and 2015–2016 were analyzed. The mean DMFT score was 10.34 (SD = 8.14), with a 33.2% prevalence of untreated dental caries. The participants had a mean age of 35.33 years (SD = 23.30), and females comprised 50.2% of the sample. The average intake of added sugars was 72.67 g (SD = 64.72).

Table 1 presents the bivariable analysis of untreated dental caries, revealing significant associations with all examined demographic factors, including the PIR and BMI. The findings indicate higher occurrences of untreated dental caries in males (34.4%), among Mexican American and Non-Hispanic Black participants (36.7%), in individuals from lower socioeconomic backgrounds (62.6%), in children of parents with a lower educational level (specifically those who completed 9th to 11th Grade, at 39.3%), and in participants with a higher BMI (39.8%).

Table 1 also reports the DMFT scores, showing higher averages in females (mean = 10.46, SD = 7.99). Significant differences were observed across the race/ethnicity, family educational level, and BMI categories. Notably, Non-Hispanic White participants (mean = 12.01, SD = 8.57) and those with parents who had lower educational levels (9th–11th Grade: mean = 11.43, SD = 8.77) and participants with the highest BMI (mean = 11.52, SD = 8.29) recorded greater DMFT scores. Following significant findings from the omnibus test, the post hoc test demonstrated significant differences in DMFT scores between all groups pairwise except the Mexican American and Non-Hispanic Asian groups (*p* > 0.05), the Mexican American and Other Race groups (*p* > 0.05), the Non-Hispanic Asian and Other Race groups (*p* > 0.05), the Other Hispanic and Non-Hispanic Black groups (*p* > 0.05), the Less than 9th Grade and Some College groups (*p* > 0.05), the High-School Graduate or GED and Less than 9th Grade groups (*p* > 0.05), the High-School Graduate or GED and Some College groups (*p* > 0.05), the Less than 9th Grade and 9th–11th Grade groups (*p* > 0.05), and the High-School Graduate or GED and Less than 9th Grade groups (*p* > 0.05).

Table 2 illustrates the distribution of serum 25(OH)D concentrations among the Americans over one year of age, showing varying deficiency levels: <25 nmol/L at 3.5%, 25–50 nmol/L at 26.8%, 50–75 nmol/L at 40.8%, and >75 nmol/L at 28.9%. Females showed higher frequencies of serum 25(OH)D concentrations below 25 nmol/L (4.0%, compared to 3.0% in males) and within the 25–50 nmol/L range (27.4%, compared to 26.2% for males), but lower frequencies in the 50–75 nmol/L category. Nonetheless, these differences lacked statistical significance across all categories. Non-Hispanic White individuals exhibited the lowest frequencies of serum 25(OH)D levels below 25 nmol/L (1.0%) and 25–50 nmol/L (12.3%, *p* < 0.001), while Non-Hispanic Black individuals had the highest (8.3% and 42.7%, respectively, *p* < 0.001). Low-income Americans were most likely to have the low serum 25(OH)D concentrations, whereas high-income individuals displayed the lowest serum 25(OH)D concentrations. Conversely, the lowest frequencies of serum 25(OH)D levels <25 nmol/L and 25–50 nmol/L were observed in the Americans with a BMI <18.5 (1.5% and 16.7%, respectively; *p* < 0.001), with the opposite trend being noted in those with a BMI >25. Following significant findings from the omnibus test, the post-hoc test revealed significant differences in serum 25(OH)D concentrations between all groups pairwise except the Mexican American and Non-Hispanic Asian groups (*p* > 0.05), the Other Hispanic and Non-Hispanic Asian groups (*p* > 0.05), the Less than 9th Grade and 9th–11th Grade groups (*p* > 0.05), and the High-School Graduate or GED and 9th–11th Grade groups (*p* > 0.05).

Table 3 explores the relationships between vitamin D levels and dental caries outcomes. Initial analyses revealed that individuals with severe vitamin D deficiency were 2.22 times (95% CI: 1.88, 2.62) more likely to have untreated dental caries compared to those with sufficient vitamin D levels. This association slightly strengthened after adjusting for demographic factors, with an adjusted odds ratio (AOR) of 2.26 (95% CI: 1.90, 2.69). The relationship remained significant, albeit reduced, after further adjusting for added sugars and BMI, with an AOR of 1.95 (95% CI: 1.64, 2.33). Additionally, a robust correlation was found between vitamin D levels and the overall prevalence of dental caries, evidenced by an RR of 1.25 (95% CI: 1.21, 1.28).

## 4. Discussion

Several risk factors were associated with untreated dental caries, DMFT scores, and vitamin D levels, including race and ethnicity, the PIR, the family educational level, and BMI. Vitamin D plays a crucial role in regulating the body’s calcium concentrations by facilitating calcium absorption. Our study, leveraging data from the NHANES, found that individuals with severe VDD have a 2.22-times higher risk of dental caries compared to those with normal vitamin D levels. Additionally, the association between VDD and dental caries intensifies with the influence of external factors such as demographics and diet. Further research corroborates a strong correlation between low vitamin D levels and increased experience and prevalence of dental caries, including untreated cases.

Research on the relationship between vitamin D and dental caries among adults in the United States is limited. Most existing studies focus on how VDD affects primary dentition due to its critical role in odontogenesis. It remains unclear whether vitamin D is equally crucial in later life stages with permanent dentition. A recent cross-sectional study involving 4244 U.S. adults aged 20–80 years found that those with severe VDD (<25 nmol/mL) were 2.48 times more likely to develop dental caries compared to those with sufficient serum levels (≥75 nmol/mL) [16]. Another study of 1688 Korean children aged 10–12 with permanent dentition showed that those with levels less than 50 nmol/L were 1.295 times more likely to experience first-molar caries compared to those with levels above 50 nmol/L. Interestingly, when controlling for all covariates, vitamin D levels were not significantly correlated with overall dental caries but were linked to first-molar caries [26]. Additionally, a study investigating dental caries and vitamin D serum levels in children aged 1–19 years found that those aged 1–5 years with the lowest vitamin D levels were 2.55 times more likely to have untreated caries compared to children with levels ≥75 nmol/mL [42]. Our study did not restrict the age range of participants in order to provide a more comprehensive overview of the correlation between vitamin D levels and dental caries. The average age of the individuals with untreated caries in our study was 35.33 years (SD = 23.30; median = 32; range = 78), indicating significant age diversity within the sample. Thus, VDD affects a broad range of individuals, regardless of age. These findings align with previous research by Zhou et al. and Kim et al. on both adults and children.

Previous studies have shown a correlation between individuals with darker skin and a slower synthesis of vitamin D from sunlight exposure compared to those with lighter skin. In our study, the proportion of serum 25(OH)D levels below 25 and 25–50 nmol/L was lowest in Non-Hispanic White participants and highest in Non-Hispanic Black participants, which is consistent with previous findings indicating lower serum levels in Non-Hispanic Black individuals and the highest sufficiency levels in Non-Hispanic White individuals [41]. Another study examining untreated and total caries among U.S. individuals aged 2 through 19 found the highest prevalence of untreated caries in Non-Hispanic Black individuals and the lowest in Non-Hispanic Asian individuals [43]. These results suggest that vitamin D synthesis varies significantly with skin color; however, Mexican Americans also showed a similar proportion of untreated caries to the Non-Hispanic Black group. This could be due to multiple factors, including a lack of preventive education [44]. For example, one study involving 48 urban Mexican American mothers revealed significant gaps in their understanding of dental caries prevention methods, proper oral hygiene, cariogenic foods beyond candy, and the impact of baby bottles on dental health [44]. Additionally, another study found that decayed and missing teeth were more prevalent among Black people than White people, whereas the opposite was true for filled teeth. Intriguingly, our data indicated that Non-Hispanic White participants had the highest DMFT scores, suggesting that a greater access to dental services could explain the higher number of filled teeth observed in this group [45].

We observed that untreated dental caries and low vitamin D levels were most prevalent among individuals from lower socioeconomic backgrounds and least prevalent among those with a higher SES. Previous studies have demonstrated that untreated dental caries is three times more common in adults over 65 living in poverty compared to those with incomes above 200% of the poverty threshold [46]. Additionally, another study found that adults with a PIR between 0.5 and 1.0, or even below 0.5, had the highest prevalence of untreated caries [47]. Recent shifts toward the early detection and prevention of dental caries aim to facilitate minimally invasive interventions [48]. However, access to dental care remains limited for poorer families, with barriers including work absence, travel costs, and childcare logistics [49]. Moreover, research in the United States has consistently shown a positive correlation between vitamin D intake and income levels [50,51]. Those living in poverty tend to have lower vitamin D levels [52]. Our findings align with these studies, indicating that SES significantly affects both the incidence of untreated dental caries and vitamin D levels. This suggests that individuals from lower socioeconomic backgrounds face a heightened risk of developing carious lesions and are likely to have insufficient vitamin D levels.

Our findings reveal that individuals with lower family education levels exhibited higher rates of untreated dental caries and DMFT scores. Conversely, those whose parents attained a college degree or higher displayed the lowest frequencies of severe VDD, moderate VDD, and insufficiency, and instead, had the highest frequencies in the sufficient category. Prior research corroborates these observations, indicating that higher parental education is associated with a lower caries prevalence and lower DMFT scores. In contrast, those from less educated backgrounds tend to have higher rates of both caries prevalence and DMFT scores [53]. Additionally, studies suggest that parents with higher education levels prioritize oral hygiene and dental checkups more than those with less education [54]. This discrepancy likely contributes to the lower vitamin D levels among less educated populations, as they may not regularly purchase foods rich in vitamin D or undergo annual health checkups to monitor for VDD [55]. Another study supports this, showing that higher parental education leads to increased vitamin D intake and reduces the likelihood of VDD and insufficiency [56]. Our data align with these findings, showing clear trends of higher untreated caries and DMFT scores, along with lower vitamin D levels, among those with lower educational backgrounds [54]. These results highlight the need for enhanced dental education and intervention, particularly for families from lower socioeconomic and educational backgrounds.

These findings underscore the importance of comprehensive patient assessments and the identification of risk factors for dental caries. Dentists must be familiar with these risk factors to effectively assist patients from diverse backgrounds. This includes providing nutritional coaching, particularly for individuals with VDD and those whose lifestyles may increase their risk of dental caries. Additionally, the consideration of socioeconomic status and family income is crucial, as these factors significantly influence a patient’s ability to maintain oral hygiene and attend regular checkups. Incorporating these considerations will foster a more patient-centered approach to dental care, ultimately elevating the standard of treatment for all patients.

While these findings are important, certain limitations should be noted. This study’s cross-sectional design limits our ability to establish cause-and-effect relationships. Large sample sizes can amplify minor statistical differences, potentially resulting in findings that, while statistically significant, may have limited clinical or operational relevance. Statistical significance indicates whether an observed effect is likely to occur due to chance, but it does not necessarily mean that this effect is meaningful or substantial in real-world settings. Therefore, it is crucial to interpret statistical outcomes with caution, considering both their clinical and practical implications. Additionally, the data sourced from the NHANES pertain solely to the United States, meaning that these findings may not be applicable to different geographic regions. Survey-based data are also subject to respondent bias, where the answers may be influenced by personal beliefs and experiences. Another limitation concerns the use of BMI to categorize individuals into overweight and underweight ranges. BMI, which calculates body fat using height and weight, does not account for muscle mass, bone density, or sex differences, which can lead to inaccuracies.

Furthermore, there are several other factors influencing dental caries that this study did not address, such as the frequency and technique of brushing and flossing, fluoride intake levels, alcohol consumption, smoking habits, and the presence of diabetes. Additionally, the integration of toothpastes containing vitamin D and hydroxyapatite, which have been studied for their effectiveness in reducing gingival inflammation and bleeding, should also be evaluated using the DMFT index [57]. For a more comprehensive understanding of the factors contributing to dental caries, future research should include these variables.

## 5. Conclusions

Our study underscores a significant correlation between vitamin D levels and the prevalence of dental caries within the United States population. Notably, lower levels of vitamin D correspond with elevated rates of untreated dental caries and increased DMFT scores. Importantly, this relationship persists even after adjusting for sociodemographic factors, indicating a direct influence of vitamin D levels on the risk of dental caries. Individuals with insufficient vitamin D face a heightened likelihood of experiencing dental caries compared to those with adequate levels. In light of these findings, it is imperative to emphasize proactive measures to maintain adequate vitamin D levels. This may involve dietary adjustments, supplementation, or the use of dental products aimed at bolstering vitamin D intake, thereby serving as a preventive strategy against dental caries.

## Figures and Tables

**Table 1:** Prevalence of untreated caries and DMFT score according to sociodemographic variables in the NHANES 2011–2016 cycles.

Variables	*n (*%)	Untreated Caries		DMFT		
		*n* (%)	*p*-Value	Mean (SD)	Median (Range)	*p*-Value
Total	18,683 (100)	6205 (33.20)	N/A	10.34 (8.14)	8 (27)	N/A
Sex						
Female	9382 (50.20)	3007 (32.10)	0.02 *	10.46 (7.99)	8 (27)	**<0.05** ^a^
Male	9301 (49.80)	3198 (34.40)		10.21 (8.29)	8 (27)	
Race and Ethnicity						
Mexican American	3042 (16.30)	1117 (36.70)	<0.05 *	7.81 (6.68)	6 (27)	**<0.05** ^b^
Other Hispanic	1983 (10.60)	657 (33.10)		10.30 (8.07)	8 (27)	
Non-Hispanic White	6720 (36.00)	2169 (32.30)		12.01 (8.57)	10 (27)	
Non-Hispanic Black	4325 (23.10)	1587 (36.70)		10.31 (8.15)	8 (27)	
Non-Hispanic Asian	1808 (9.70)	449 (24.80)		8.31 (6.85)	6 (27)	
Other Race ^#^	805 (4.30)	226 (28.10)		9.14 (8.17)	6 (27)	
PIR						
<1.3	7019 (37.60)	2625 (62.60)	<0.05 *	10.51 (8.60)	8 (27)	0.83 ^b^
1.3–3.5	6741 (36.10)	2300 (34.10)		10.38 (8.26)	8 (27)	
>3.5	4923 (26.4)	1280 (26.0)		10.01 (7.21)	9 (27)	
Family Educational Level						
<9th Grade	1694 (9.10)	615 (36.30)	<0.05 *	10.93 (9.04)	8 (27)	**<0.05** ^b^
9th–11th Grade	2492 (13.30)	979 (39.30)		11.43 (8.77)	9 (27)	
High-School Graduate or GED	4110 (22.00)	1565 (38.10)		10.77 (8.34)	8 (27)	
Some College	5734 (30.70)	1895 (33.00)		10.17 (7.96)	8 (27)	
College Graduate or Higher	4653 (24.90)	1151 (24.70)		9.19 (7.17)	7 (27)	
Body Mass Index						
<18.5	3087 (16.50)	543 (17.60)	<0.05 *	5.40 (5.30)	4 (27)	**<0.05** ^b^
18.5–25	5759 (30.80)	1742 (30.20)		9.46 (7.85)	7 (27)	
>25	9837 (52.70)	3920 (39.80)		11.52 (8.29)	10 (27)	

Note: Bold indicates *p* < 0.05. ^#^ Includes multi-racial participants. * Chi-square test. ^a^ Mann–Whitney U test. ^b^ Kruskal–Wallis test.

**Table 2:** Prevalence of vitamin D according to sociodemographic variables in the NHANES 2011–2016 cycles.

Variables	*n*(%)	<25 nmol/L *n* (%)	25–50 nmol/L *n* (%)	50–75 nmol/L *n* (%)	>75 nmol/L *n* (%)	*p*-Value
Total	18,683 (100)	649 (3.50)	5006 (26.80)	7624 (40.80)	5404 (28.90)	
Sex						
Female	9382 (50.20)	372 (4.00)	2566 (27.40)	3560 (37.90)	2884 (30.70)	0.30 ^a^
Male	9301 (49.80)	277 (3.00)	2440 (26.20)	4064 (43.70)	2520 (27.10)	
Race and Ethnicity						
Mexican American	3042 (16.30)	99 (3.30)	1002 (32.90)	1476 (48.50)	465 (15.30)	**<0.05** ^b^
Other Hispanic	1983 (10.60)	47 (2.40)	536 (27.00)	983 (49.60)	417 (21.00)	
Non-Hispanic White	6720 (36.00)	64 (1.00)	824 (12.30)	2640 (39.30)	3192 (47.50)	
Non-Hispanic Black	4325 (23.10)	361 (8.30)	1845 (42.70)	1450 (33.50)	669 (15.50)	
Non-Hispanic Asian	1808 (9.70)	59 (3.30)	627 (34.70)	712 (39.40)	410 (22.70)	
Other Race ^#^	805 (4.30)	19 (2.40)	172 (21.40)	363 (45.10)	251 (31.20)	
PIR						
<1.3	7019 (37.60)	300 (4.30)	2192 (31.20)	2969 (42.30)	1558 (22.20)	**<0.05** ^b^
1.3–3.5	6741 (36.10)	239 (3.50)	1825 (27.10)	2744 (40.70)	1933 (28.70)	
>3.5	4923 (26.40)	110 (2.20)	989 (20.10)	1911 (38.80)	1913 (38.90)	
Family Educational Level						
<9th Grade	1694 (9.10)	48 (2.80)	546 (32.20)	764 (45.10)	336 (19.80)	**<0.05** ^b^
9th–11th Grade	2492 (13.30)	101 (4.10)	764 (30.70)	1053 (42.30)	574 (23.00)	
High-School Graduate or GED	4110 (22.00)	157 (3.80)	1191 (29.00)	1693 (41.20)	1069 (26.00)	
Some College	5734 (30.70)	225 (3.90)	1514 (26.40)	2292 (40.00)	1703 (29.70)	
College Graduate or Higher	4653 (24.90)	118 (2.50)	991 (21.30)	1822 (39.20)	1722 (37.00)	
Body Mass Index						
<18.5	3087 (16.50)	45 (1.50)	516 (16.70)	1570 (50.90)	956 (31.00)	**<0.05** ^b^
18.5–25	5759 (30.80)	163 (2.80)	1512 (26.30)	2303 (40.00)	1781 (30.90)	
>25	9837 (52.70)	441 (4.50)	2978 (30.30)	3751 (38.10)	2667 (27.10)	

Note. Bold indicates *p* < 0.05. ^#^ Includes multi-racial participants. ^a^ Mann–Whitney U test. ^b^ Kruskal–Wallis test.

**Table 3:** Models examining the relationships between vitamin D levels and both the presence of caries and the presence of untreated caries.

Variables	*n* (%)	Model 1 ^1^	Model 2 ^2^	Model 3 ^3^
		OR [95% CI]	OR [95% CI]	OR [95% CI]
Untreated Caries				
>75 nmol/L (sufficiency)	1598 (25.80)	Reference	Reference	Reference
50–75 nmol/L (insufficiency)	2428 (39.10)	1.11 [1.03–1.20] *	1.28 [1.18–1.39] *	1.21 [1.11–1.31] *
25–50 nmol/L (moderate deficiency)	1866 (30.10)	1.42 [1.30–1.54] *	1.53 [1.40–1.68] *	1.36 [1.24–1.50] *
<25 nmol/L (severe VDD)	313 (5.00)	2.22 [1.88–2.62] *	2.26 [1.90–2.69] *	1.95 [1.64–2.33] *
	***n*** **(%)**	**RR [95% CI]**	**RR [95% CI]**	**RR [95% CI]**
Caries				
>75 nmol/L (sufficiency)	4002 (30.10)	Reference	Reference	Reference
50–75 nmol/L (insufficiency)	5170 (38.80)	0.95 [0.93–0.98] *	1.08 [1.07–1.09] *	1.07 [1.06–1.08] *
25–50 nmol/L (moderate deficiency)	3615 (27.20)	0.78 [0.76–0.79] *	1.14 [1.12–1.16] *	1.11 [1.10–1.13] *
<25 nmol/L (severe VDD)	523 (3.90)	0.73 [0.72–0.74] *	1.29 [1.25–1.33] *	1.25 [1.21–1.28] *

^1^ Model 1 was not adjusted. ^2^ Model 2 was adjusted for demographic factors (sex, age, race/ethnicity, PIR, family educational level). ^3^ Model 3 was additionally adjusted for the intake of added sugars and BMI. * *p* < 0.05.

## Data Availability

Data are available at https://wwwn.cdc.gov/nchs/nhanes/Default.aspx (accessed on 25 July 2023).
